# O019: A new genre of surface disinfectant with long residual bactericidal activity

**DOI:** 10.1186/2047-2994-2-S1-O19

**Published:** 2013-06-20

**Authors:** A Sava, S Kritzler

**Affiliations:** 1 Research, Novapharm Research Australia, Sydney, Australia

## Introduction

Whilst antibacterial copper is regarded as effective for continuous antibacterial protection in high traffic areas, high cost and poor efficacy against some microorganisms restricts its application.

Evocide is a new genre of liquid surface disinfectant with broad spectrum antibacterial efficacy on application. Unlike conventional surface disinfectants, it dries to leave an invisible fingerprint-and water-resistant film that continues killing bacteria on contact for up to six months. These films readily dissolve during subsequent applications of Evocide or in >20% ethanol.

We present results of assaying the Evocide’s bactericidal efficacy both as liquid and as dried films.

## Methods

The bactericidal properties of liquid disinfectant were assayed as per TGO 54 and relevant AOAC bactericidal protocols.

The bactericidal properties of residual films were tested as per the US EPA’s self-sanitising surface test, where the residual films are inoculated after alternating wet and dry abrasions to simulate fingerprint traffic.

## Results

On application >6log reduction of bacterial inocula is achieved within 2 minutes for *S. aureus*, *E. coli*, *P. aeruginosa* and within 5 minutes for *M. bovis* (TB).

The residual film, even after 10 wet and 10 dry abrasions, reduces the population of *E. coli* and *Klebsiella pneumoniae* by >3-log within 10 min after inoculation (compared to 2 hours for antimicrobial copper). The abraded residual films also achieve >3-log reduction of *P.aeruginosa* and vegetative *C. difficile* inocula within 10 minutes. Surprisingly, the films also exhibit some sporicidal properties with the counts of spore suspensions of *C.difficile* and *B.subtilis* being reduced by 40% and 70% respectively within 24 hours after inoculation.

Evocide also complies with the relevant criteria for toxicity, biocompatibility and no-film build-up.

## Conclusion

This new genre of surface disinfectant exhibits powerful residual bactericidal properties combined with good materials compatibility. It allows for the creation of a potent antimicrobial barrier on any environmental hard surface at fraction of the cost of copper. It also offers a simple safeguard for cleaning deficiencies, ensuring that hard-to-reach surfaces (eg beds, bedside tables) remain hygienic.

## Disclosure of interest

None declared.

